# Neutrophils Reduce the Parasite Burden in *Leishmania (Leishmania) amazonensis*-Infected Macrophages

**DOI:** 10.1371/journal.pone.0013815

**Published:** 2010-11-03

**Authors:** Érico Vinícius de Souza Carmo, Simone Katz, Clara Lúcia Barbiéri

**Affiliations:** Departamento de Microbiologia, Imunologia e Parasitologia, Universidade Federal de São Paulo, São Paulo, Brasil; University of California, San Francisco, United States of America

## Abstract

**Background:**

Studies on the role of neutrophils in *Leishmania* infection were mainly performed with *L. (L) major*, whereas less information is available for *L. (L) amazonensis*. Previous results from our laboratory showed a large infiltrate of neutrophils in the site of infection in a mouse strain resistant to *L. (L.) amazonensis* (C3H/HePas). In contrast, the susceptible strain (BALB/c) displayed a predominance of macrophages harboring a high number of amastigotes and very few neutrophils. These findings led us to investigate the interaction of inflammatory neutrophils with *L. (L.) amazonensis*-infected macrophages *in vitro*.

**Methodology/Principal Findings:**

Mouse peritoneal macrophages infected with *L. (L.) amazonensis* were co-cultured with inflammatory neutrophils, and after four days, the infection was quantified microscopically. Data are representative of three experiments with similar results. The main findings were 1) intracellular parasites were efficiently destroyed in the co-cultures; 2) the leishmanicidal effect was similar when cells were obtained from mouse strains resistant (C3H/HePas) or susceptible (BALB/c) to *L. (L.) amazonensis*; 3) parasite destruction did not require contact between infected macrophages and neutrophils; 4) tumor necrosis factor alpha (TNF-α), neutrophil elastase and platelet activating factor (PAF) were involved with the leishmanicidal activity, and 5) destruction of the parasites did not depend on generation of oxygen or nitrogen radicals, indicating that parasite clearance did not involve the classical pathway of macrophage activation by TNF-α, as reported for other *Leishmania* species.

**Conclusions/Significance:**

The present results provide evidence that neutrophils in concert with macrophages play a previously unrecognized leishmanicidal effect on *L. (L.) amazonensis*. We believe these findings may help to understand the mechanisms involved in innate immunity in cutaneous infection by this *Leishmania* species.

## Introduction

Neutrophils, key players of the innate immune system, provide a first line of defense against invading pathogens. Neutrophils may be also implicated in immunoregulation as a source of cytokines, such as interleukin-2 (IL-12), interleukin-10 (IL-10), gamma interferon (IFN-γ) and TNF-α [1], [2], thus establishing a link between innate and adaptative immunity during parasitic infection [3], [4]. Studies have shown that neutrophils could protect or enhance infection with *Leishmania (Leishmania) major* [5]–[8]. Neutrophils were also shown to behave like Trojan horses, sponsoring the invasion of macrophages by *L. (L.) major in vitro* [9] and *in vivo* [10]. A role for neutrophil in protective immune responses to *L. (Viannia) braziliensis* [11] and to visceralizing *Leishmania* species was also reported [12]–[15].

In previous studies neutrophils were detected in *L. (L.) amazonensis* lesions soon after infection [16]. Neutrophils were also implicated in chemotactic responses to *L. (L.) amazonensis* promastigotes, in the destruction of these parasites and in the release of leishmanicidal effectors [17]–[19]. More recently, human apoptotic and necrotic neutrophils were shown to increase and to reduce, respectively, *L. (L.) amazonensis* parasite burden in infected macrophages [20].

We have previously observed that neutrophils predominate at the sites of infection with *L. (L.)* amazonensis in resistant C3H/HePas mice which displayed a low parasite burden. In contrast, few neutrophils were found in the parasite-rich lesions of susceptible BALB/c mice (unpublished results). These observations suggest that neutrophils could play a role in the resistance of C3H/HePas mice to the parasite. In the present study we investigated the *in vitro* interaction of neutrophils with *L. (L.) amazonensis*-infected macrophages. We report that *L. (L.) amazonensis* amastigotes were destroyed when infected peritoneal macrophages from either susceptible BALB/c or resistant C3H/HePas mice were co-cultured with syngeneic inflammatory neutrophils. The leishmanicidal activity did not require cell to cell contact and was mediated by TNF-α, neutrophil elastase and platelet activating factor. These findings indicate that inflammatory neutrophils may play a role in innate host defense against *L. (L.) amazonensis*.

## Results

### *L. (L.) amazonensis* amastigotes are killed after addition of neutrophils to infected macrophages

**Figure 1:**
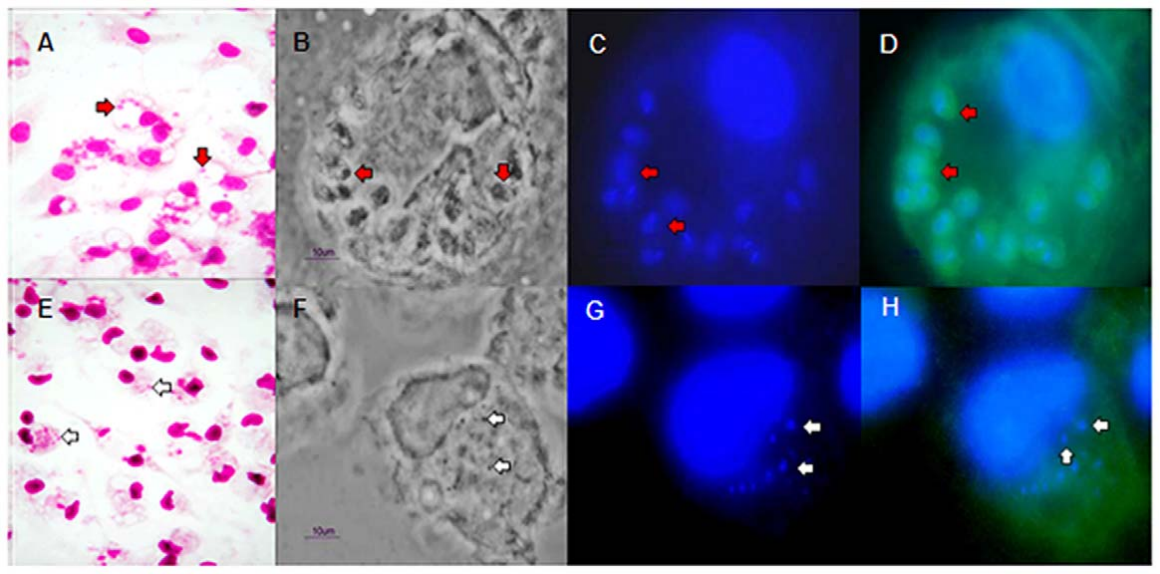
Light microscopy and immunofluorescence of neutrophils co-cultured with *L. (L.) amazonensis*-infected macrophages. *L. (L.) amazonensis*-infected macrophages were either cultured in the absence (upper sequence) or in the presence of inflammatory neutrophils (lower sequence). After 4 days the cultures were stained with HE (A, E) or DAPI (C, G) or incubated with a rabbit serum anti-*L. (L.) amazonensis* amastigotes and stained with DAPI (D, H). B, F, Nomarski interference contrast. Red arrows indicate intact amastigotes; white arrows show parasite debris. A, E – Magnification, x1,000. B, C, D, F, G, H - Magnification, x400.

Inflammatory neutrophils isolated from peritoneal cavities of BALB/c mice 7 h after they had received an intraperitoneal injection of starch were co-cultured for four days with mouse peritoneal macrophages previously infected with *L. (L.) amazonensis*. The macrophage infection was followed by light microscopy and immunofluorescence. Destruction of amastigotes could be observed by the presence of parasite debris inside *L. (L.) amazonensis*-infected macrophages co-cultured with neutrophils, as indicated by the arrows in Figure 1, E to H. In contrast, healthy amastigotes could be visualized in control cultures (arrows in Figure 1, A to D).

### Effect of neutrophils on the infection of macrophages from mouse strains susceptible or resistant to *L. (L.) amazonensis*

**Figure 2:**
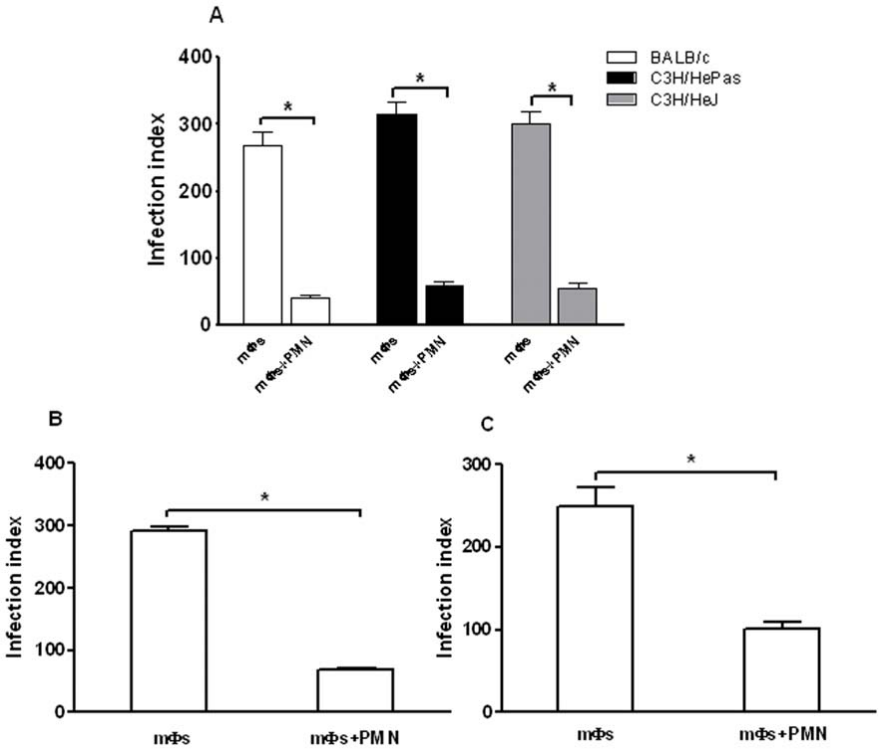
Parasite loads in *L. (L.) amazonensis*-infected macrophages co-cultured with neutrophils from BALB/c, C3H/HePas and C3H/HeJ mice. (A) Inflammatory neutrophils from either BALB/c, C3H/HePas or C3H/HeJ mice were co-cultured with syngeneic *L. (L.) amazonensis*-infected macrophages (10∶1) in a same chamber. Inflammatory neutrophils from BALB/c (B) or C3H/HePas mice (C) were co-cultured with syngeneic *L. (L.) amazonensis*-infected macrophages in transwell plates. After four days of incubation, cells were fixed and stained for parasite counts. Bars represent the SD. ^*^ *P*<0.001.

In these experiments, macrophages and syngeneic neutrophils were obtained from either susceptible BALB/c or resistant C3H/HePas and C3H/HeJ mice previously activated with starch, as described in Materials and Methods. Figure 2A shows that infection of macrophages was significantly decreased in the presence of neutrophils in comparison to controls and that parasite destruction was similar in co-cultures of the three mouse strains.

### Neutrophil-macrophage contact is not required for leishmanicidal activity

A transwell system (0.4 µm) was used to determine if cell contact was required for leishmanicidal activity in the co-cultures. Figures 2B and 2C show that the leishmanicidal effect was similar whether cells were separated by a cell-impermeable membrane or cultivated together. These results indicate that the leishmanicidal activity is mediated by soluble factor (s) released in the cultures.

### Cytokine secretion in co-cultures of neutrophils and *L. (L.) amazonensis*-infected macrophages

**Figure 3:**
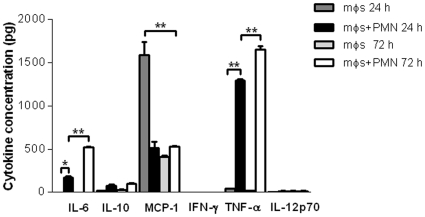
Cytokine secretion in supernatants from the co-cultures of neutrophil/*L. (L.) amazonensis*-infected macrophages. Inflammatory neutrophils from BALB/c mice were co-cultured with *L. (L.) amazonensis*-infected macrophages (10∶1). Supernatants from 24 and 72 h cultures were tested at 1∶2 dilution for Cytometric Bead Array immunoassay as described in Section Materials and Methods. Cytokine concentrations were determined from standard curves plotted using a four-parameter logistic curve fitting model. A value of 0 was assigned when the concentration of a cytokine was below the detection limit for the assay. Bars represent the SD. *P<0.05 and **P<0,001.

To estimate the concentrations of cytokines released into the co-culture medium, concentrations of interleukin-6 (IL-6), TNF-α, IFN-γ, monocyte chemotactic protein-1 (MCP-1), IL-12p70 and IL-10 were determined in 24 and 72 h supernatants by a Cytometric Bead Array (see Materials and Methods). Figure 3 shows that, in the absence of added neutrophils, a significantly higher concentration of MCP-1 was found in the supernatants of *L. (L.) amazonensis*-infected macrophages. Only very low concentrations of IL-10, IFN-γ and IL-12 were detected in supernatants of infected macrophages and co-cultures. On the other hand, high concentrations of TNF-α and IL-6 were detected in the co-cultures and both peaked at 72 h. Taken together, these results indicate that an inflammatory-like environment predominates in the co-cultures.

### Effect of TNF-α on parasite killing in the co-cultures

**Figure 4:**
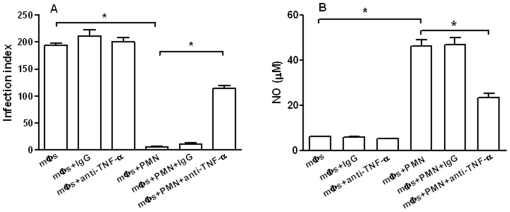
Anti-TNF-α inhibited the leshmanicidal activity in *L. (L.) amazonensis* infected macrophages co-cultured with neutrophils. The co-cultures were maintained in the presence of a monoclonal anti-TNF-α for four days and cells were fixed and stained for infection index determination (A). Nitric oxide was assayed in the co-cultures supernatants (B). Bars represent the SD. ^*^ *P*<0.001.

The demonstration that TNF-α was secreted in the medium of neutrophil/L. (L.) amazonensis-infected macrophage co-cultures led us to evaluate the participation of this cytokine in parasite destruction. Figure 4 shows that anti-TNF-α added to the cultures reverted the amastigote killing by about 30% and significantly decreased nitric oxide (NO) secretion. These results indicate that TNF-α is required for the leishmanicidal activity and that NO could be associated with parasite destruction.

### Role of NO and oxygen radicals in neutrophil mediated leishmanicidal activity

**Figure 5:**
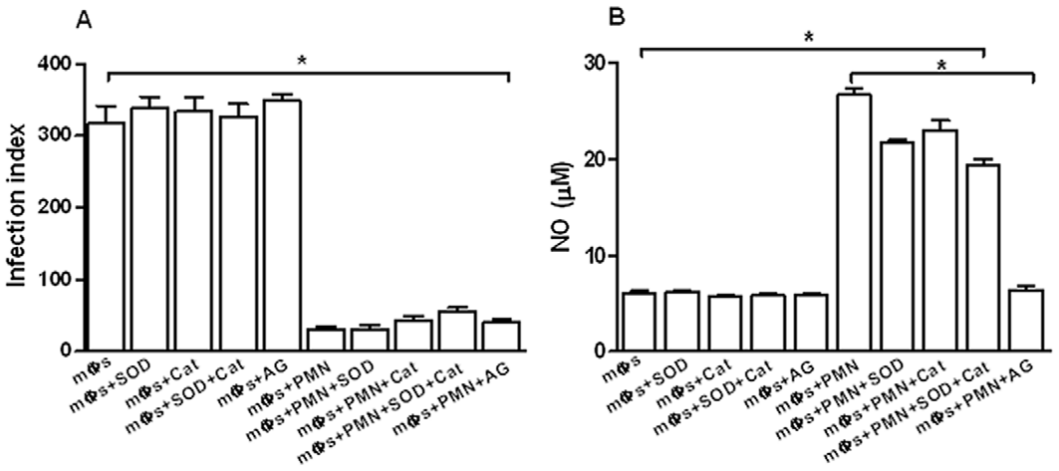
Effect of NO, superoxide anion and hydrogen peroxide depletion on parasite load in *L. (L.) amazonensis*-infected macrophages co-cultured with neutrophils (A) and secretion of nitric oxide in the supernatants from these co-cultures (B). Bars represent the SD. ^*^ *P*<0.001.

The findings that TNF-α is involved with the leishmanicidal activity and that NO is released in the co-cultures led us to evaluate the requirement for nitrogen and oxygen radicals in L. (L.) amazonensis destruction. Figure 5 shows that, although the secretion of NO in the co-culture supernatants was decreased by aminoguanidine, the inhibitor did not affect amastigote destruction (Figure 5). Similar results were obtained when superoxide dismutase (SOD) and catalase, two respiratory burst scavengers, were tested in the co-cultures. These findings indicate that L. (L.) amazonensis killing is independent of NO and respiratory burst. Thus, the effect of TNF- α on parasite killing may not be related to classic macrophage activation.

### Effect of TNF-α on parasite burden in *L. (L.) amazonensis*-infected macrophages

**Figure 6:**
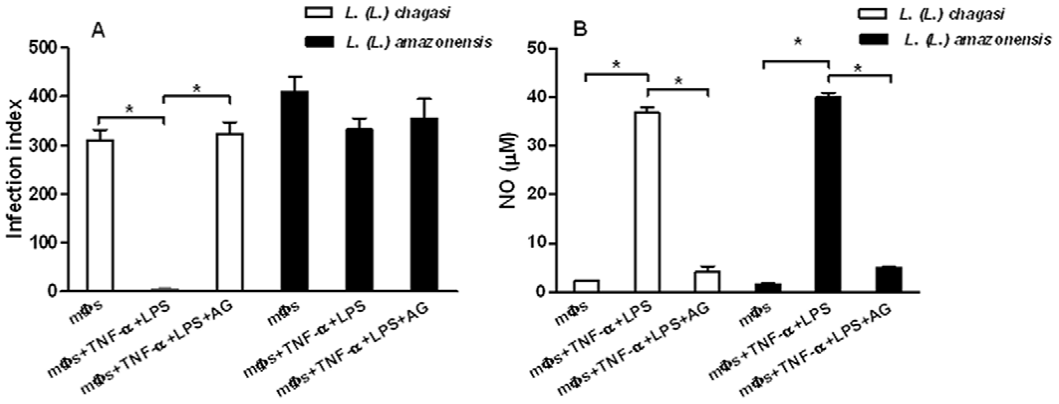
Activation of *L. (L.) amazonensis* and *L*. (*L*.) *chagasi* infected macrophages. Infected macrophages were cultured in the presence of TNF-α plus LPS. Four days later, cultures were fixed and stained for infection index determination. *L*. (*L*.) *chagasi* was used as a positive control for activation (A). Nitric oxide was assayed in the culture supernatants (B). Bars represent the SD. ^*^ *P*<0.001.

To confirm that activation of macrophages by TNF-α is not necessarily associated with the leishmanicidal activity, BALB/c peritoneal macrophages infected with *L. (L.) amazonensis* were activated with TNF-α plus lipopolysaccharide (LPS). As a positive control BALB/c macrophages infected with *L. (L.) chagasi* were similarly activated. Figure 6 shows that *L. (L.) chagasi*-infected macrophages activated with TNF-α produced a high concentration of NO (Figure 6B) followed by a significant leishmanicidal activity which could be reverted by the addition of aminoguanidine (Figure 6A). In contrast, *L. (L.) amazonensis* amastigotes were not destroyed by the activated macrophages (Figure 6A).

### Effect of neutrophil elastase and platelet activating factor on parasite killing in the co-cultures

**Figure 7:**
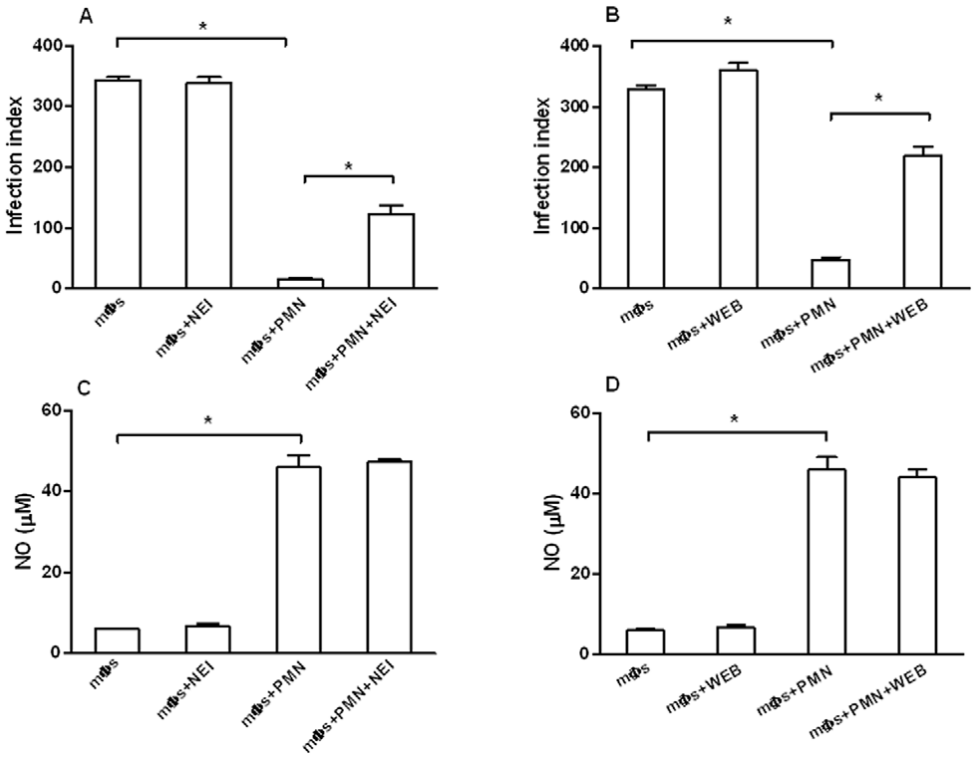
Effect of NE inhibitor (A), and PAF antagonist (B) on the leishmanicidal activity in *L. (L.) amazonensis*-infected macrophages co-cultured with neutrophils and secretion of nitric oxide in the supernatants from these co-cultures (C and D). Bars represent the SD. ^*^ *P*<0.001.

Neutrophil elastase was shown to be involved in parasite killing when dead neutrophils were co-cultured with macrophages infected with *L. (L.) major* or *L. (L.) amazonensis* [7], [20]. Leishmanicidal activity of PAF on *L. (L.) amazonensis in vitro* and *in vivo* infection was also previously reported [21], [22]. These findings led us to test the effect of neutrophil elastase and PAF on parasite destruction in the co-cultures. Figure 7 shows that the specific neutrophil elastase inhibitor methoxysuccinyl-Ala-Ala-Pro-Val-chloromethylketone (MeOSuc-AAPV-CMK) or the PAF antagonist 3-[4-(2-chlorophenyl)-9-methyl-6*H*-thieno[3,2-*f*][1], [2], [4]triazolo[4,3-*a*][1], [4]-diazepine-2-yl]-1-(4-morpholinyl)-1-propanone (WEB-2086) significantly reduced neutrophil mediated leishmanicidal activity. A significant concentration of NO was detected in the supernatants of the co-cultures and its secretion was unaffected by the two inhibitors, suggesting that NO is not implicated in the leishmanicidal acitivity induced by elastase or PAF.

## Discussion

We have shown that murine *L. (L.) amazonensis*-infected macrophages co-cultured with neutrophils destroyed the intracellular amastigotes (Figures 1 and 2). The leishmanicidal activity did not depend on the host resistance profile since no significant difference in parasite destruction was observed between co-cultures from *L. (L.) amazonensis* susceptible and resistant mice (Figure 2). It was previously reported that live neutrophils from resistant and susceptible mice were equally efficient at inducing parasite destruction when co-cultured with *L. (V.) braziliensis*-infected macrophages [11]. In the same study, the authors noted that addition of live neutrophils to macrophages infected with *L. (L.) amazonensis* led to a significant reduction in parasite loads. However, mechanisms involved in the neutrophil mediated leishmanicidal activity on *L. (L.) amazonensis* were not investigated [11].

Cytokine analyses showed that MCP-1 was only detected in supernatants from *L. (L.) amazonensis*-infected macrophages at 24 h and its secretion was very low at 72 h when macrophage infection was well-established (Figure 3). This observation agrees with studies that showed a rapid and transient MCP-1 secretion, as well as MCP-1 gene expression in macrophages infected with different *Leishmania* species [23]–[25]. Although it was known that MCP-1 reduced parasite burden in *L. (L.) amazonensis*-infected macrophages [26], in our experiments the low levels of MCP-1 detected in the co-cultures suggest that this cytokine is not required for the leishmanicidal activity. However, an inflammatory environment mediated by IL-6 and TNF-α was detected in the co-cultures (Figure 3) and the significant inhibition of parasite killing by anti-TNF-α indicated the involvement of this cytokine in parasite destruction (Figure 4). These findings corroborate those reported for *L. (L.) amazonensis*-infected human macrophages co-cultured with necrotic neutrophils [20]. However, whereas in that study necrotic neutrophils were phagocytosed by the infected macrophages [20], in our work live neutrophils were co-cultured with *L. (L.) amazonensis*-infected macrophages and parasite killing could take place in the absence of cell contact (Figure 2). Furthermore, in that study *L. (L.) amazonensis* destruction was dependent on superoxide, whereas in our experiments inhibition of oxygen radicals secretion did not revert the leishmanicidal activity (Figure 5). Our findings also differ from those reported for co-cultures of *L. (V.) braziliensis*-infected macrophages and live neutrophils, since in that study parasite killing required both cell contact and production of superoxide [11]. The discrepancy between our data and those observed in co-cultures of *L. (V.) braziliensis*-infected macrophages and live neutrophils are possibly due to the genetic and biological diversity between *L. (L.) amazonensis* and *L. (V.) braziliensis* that determines the differences in outcome of cutaneous leishmaniasis caused by these two species.

We also found that inhibition of NO production did not revert leishmanicidal activity (Figure 5). These findings were strengthened by the observation that *L. (L.) amazonensis* amastigotes were not destroyed by infected macrophages stimulated with TNF-α plus LPS in spite of a significant NO secretion (Figure 6). Taken together, these results indicate that the leishmanicidal activity of TNF-α in the co-cultures was not due to classical macrophage activation by this cytokine which was previously shown to induce destruction of *L. (L.) major* and *L. (L.) donovani* through the production of oxygen radicals and nitric oxide [27], [28]. Indeed, our findings underline the singular features of the interaction of *L. (L.) amazonensis* with host macrophages.

Besides TNF-α, we have also shown that neutrophil elastase, as well as PAF, mediate destruction of *L. (L.) amazonensis* (Figure 7). A role for neutrophil elastase in leishmanicidal activity was previously reported in *L. (L.) amazonensis* and *L. (L.) major* models. Thus, this enzyme was reported to mediate *L. (L.) amazonensis* destruction in human infected macrophages co-cultured with necrotic neutrophils [20]. In another report, participation of NE in killing of *L. (L.) major* in infected macrophages co-cultured with inflammatory neutrophils required Toll-like receptor 4 (TLR4) signaling [29]. In the present studies, *L. (L.) amazonensis* destruction does not seem to require recruitment of TLR4 by neutrophil elastase since the leishmanicidal activity was independent on cell contact (Figure 2). Furthermore, *L. (L.) amazonensis* was also killed in co-cultures of C3H/HeJ mouse strain that does not express the TLR4 receptor (Figure 2). The role of elastase as an inducer of TNF-α production was demonstrated in human macrophages exposed to blood neutrophil lysates [30]; a similar mechanism could be exerted by this enzyme in our experiments since the inhibition of both, elastase and TNF-α, reverted the parasite destruction.

The involvement of PAF in the leishmanicidal activity of the co-cultures is indicated by its reversion by the PAF antagonist WEB 2086. PAF has been previously shown to inhibit *L. (L.) amazonensis* infection of mouse macrophages *in vitro* and *in vivo* in both a NO-dependent and independent fashion [21], [22]. The synthesis of PAF can be induced by TNF-α in several cell types, such as neutrophils and macrophages [31] and in a bimodal manner PAF also enhances the production of TNF-α by macrophages [32]. Although leukotrienes were reported to be involved with *L. (L.) amazonensis* killing and they can be produced by activate macrophages and neutrophils [33]–[35], we were unable to demonstrate their participation in the parasite destruction in the co-culture model used (data not shown).

We propose that a positive feedback loop established by TNF-α, neutrophil elastase and PAF may regulate the leishmanicidal activity in the co-cultures. Although both NE and TNF-α induce oxidant species in macrophages [36]–[38], in our model parasite destruction did not depend on the production of superoxide, hydrogen peroxide or nitric oxide (Figures 5 and 7). Furthermore, our findings do not exclude an additional role for other neutrophil enzymes, such as cathepsin G and myeloperoxidase in the leishmanicidal activity observed. Overall, our results are compatible with the hypothesis that transfer of neutrophil effector molecules to infected macrophages directly or indirectly mediates parasite destruction in the co-cultures.

In conclusion, the present study demonstrated that neutrophils in concert with macrophages are able to efficiently kill *L. (L.) amazonensis*. Our findings provide additional evidence for the variety of mechanisms that allows neutrophils to cooperate with macrophages in innate immunity to microbial pathogens [3].

## Materials and Methods

### Animals and parasites

Female BALB/c, C3H/HePas and C3H/HeJ mice 8 to 12 weeks old were purchased from breeding stock maintained at the University of São Paulo. Eight-week-old female golden hamsters were obtained from University of Campinas, São Paulo, Brazil. All animal procedures were approved by the Ethical Committee for Animal Care at the Federal University of São Paulo. The *L*. (*L*.) *amazonensis* (MHOM/BR/1973/M2269) and the *L*. (*L*.) *chagasi* (MHOM/BR/1972/LD) strains used were kindly provided by J. J. Shaw (Instituto Evandro Chagas, Belém, Pará, Brazil) and maintained as amastigotes in infected hamsters as previously described [39].

### Exudate neutrophils

Peritoneal exudate neutrophils were obtained 7 h after intraperitoneal injection of 0.5 ml of 10% starch (Sigma-Aldrich, St. Louis, MO) prepared in phosphate-buffered saline (PBS). Exudate cells were collected, washed, counted in a hemocytometer after staining with Diff-Quick and resuspended in complete culture medium [RPMI 1640 medium (Sigma-Aldrich) supplemented with 10% of fetal calf serum (FCS) (Gibco, Grand Island, NY), 2 mM L-glutamine, 100 U/ml penicillin and 100 µg/ml streptomycin]. In all experiments neutrophil suspensions were used immediately after their preparation.

### Inflammatory macrophages, infection and co-cultures

Inflammatory macrophages were obtained 4 days after intraperitoneal injection of 0.5 ml of 10% starch (Sigma-Aldrich) prepared in PBS. Peritoneal exudate cells (5×10^5^) were allowed to attach to round 12 mm-diameter cover glasses. These were rinsed with PBS and placed in 16 mm-diameter wells of Corning plates (Corning Glass, Corning, NY) containing 0.5 ml of RPMI with 10% heat-inactivated FCS, 100 units/ml penicillin and 100 µg/ml streptomycin, and incubated in an atmosphere of air/CO_2_ (95/5%) at 37°C. After 24 h, the macrophage cultures were infected for 4 h with *L. (L.) amazonensis* at a multiplicity of infection of 3 amastigotes per cell. Inflammatory neutrophils were added at a 10∶1 ratio (5×10^6^ cells/coverslip). In some experiments, macrophage monolayers and neutrophils were separated by a cell-impermeable culture insert membrane (Trans-Well system, 0.4 µm; Corning). In some experiments peritoneal macrophages were cultured in absence of neutrophils and infected either with *L. (L.) amazonensis* (three amastigotes per macrophage) or with *L. (L.) chagasi* (five amastigotes per cell). After 24 h, infected macrophages were activated with 200 ng/ml of TNF-α (R&D Systems, Minneapolis, MN, USA) and 10 ng/ml of LPS (Sigma-Aldrich). Co-cultures and infected macrophages were kept for 4 days at 37°C, 5% CO_2_, fixed with methanol, stained with hematoxylin-eosin (HE) and intracellular amastigotes were counted. Results are expressed by the infection index, obtained by multiplying the percentage of infected macrophages by the average number of amastigotes per macrophage. At least 200 macrophages were scored in each of 3 coverslips.

### Antibodies and reagents

Mouse anti-TNF-α or mouse immunoglobulin G (IgG) control (R&D Systems) were used at 10 µg/ml. Co-cultures were also treated with the specific neutrophil elastase inhibitor MeOSuc-AAPV-CMK (Calbiochem-Novabiochem, La Jolla, CA) at 10 µg/ml. NO synthase inhibitor aminoguanidine (Sigma-Aldrich) was used at 1 mM. The PAF antagonist WEB-2086 (Boehringer Ingelheim, Germany) was used at 10^−5^ M. Catalase and superoxide dismutase (Sigma-Aldrich) were both used at 1,000 U/ml.

### Cytokine measurement

Supernatants from either infected macrophages or neutrophil/infected macrophage co-cultures were collected after 24 and 72 h, cleared by centrifugation, and immediately assayed for IL-6, IL-10, MCP-1, IFN-γ, TNF-α and IL-12 p70 content, by Cytometric Bead Array, according to the manufacturer instructions (BD Biosciences, San Diego, CA).

### Nitric oxide production

Nitric oxide concentrations were determined by adding 100 µl of supernatants from infected cultures to 100 µl of Griess reagent [1% sulfanilamide and 0.1% N-(1-naphthyl)ethylenediamine dihydrochroride (Sigma-Aldrich) in H_3_PO_4_ 2.5%]. After incubation for 10 min at room temperature, absorbance was measured at 540 nm in a Multiskan Plate Reader (Labsystems Oy, Helsinki, Finland). Sodium nitrite (NaNO_2_) diluted in culture medium was used as standard.

### Immunofluorescence microscopy

Coverslips containing *L. (L.) amazonensis*-infected macrophages co-cultured for four days with neutrophils were washed with PBS, fixed for 1 h in 3.5% formalin in 6% of sucrose, washed in PBS containing 0.1% of bovine serum albumin (BSA) (Sigma-Aldrich), treated with 0.01% saponin (Sigma- Aldrich) for 30 min and incubated with a rabbit antibody anti-*L. (L.) amazonensis* for 1 h at room temperature. Controls were incubated with a rabbit non-specific IgG (Sigma-Aldrich). Coverslips were incubated for 30 min with fluorescein isothiocyanate-conjugated goat anti-rabbit IgG (Sigma-Aldrich) diluted in PBS-BSA/saponin, washed and incubated in 50 mM of 4′,6-diamidino-2-phenylindole (DAPI) (Molecular Probes, Eugene OR) for 30 min. After washing, the coverslips were mounted in 0.1 M glycerol and examined on a Nikon fluorescence microscope.

### Statistical analysis

One-way ANOVA and Student's *t* test were used to determine the statistical differences between groups by use of GraphPad Prism (version 5.0). Differences with a *P* value <0.05 or lower were considered significant.
